# Probing the roles of developmentally active neurons, in early-life adversity induced disruptions of adult behaviors

**DOI:** 10.3389/fnins.2025.1671495

**Published:** 2025-09-23

**Authors:** Ryan Weber, Ali Mortazavi, Tallie Z. Baram, Amalia Floriou-Servou

**Affiliations:** ^1^Department of Developmental and Cell Biology, University of California, Irvine, Irvine, CA, United States; ^2^Department of Anatomy and Neurobiology, University of California, Irvine, Irvine, CA, United States; ^3^Department of Pediatrics, University of California, Irvine, Irvine, CA, United States; ^4^Department of Neurology, University of California, Irvine, Irvine, CA, United States

**Keywords:** early life adversity, genetic tagging, TRAP, immediate early genes, transcriptomics, epigenomics

## Abstract

Early life adversity (ELA) is associated with subsequent mental health problems, and animal studies provide evidence for a causal role of ELA in the risk for mental illness, including persistent brain changes at molecular, cellular, network and functional levels. As enduring changes in cell function depend on orchestrated expression of genes, a robust body of research has focused on identifying the specific epigenetic and transcriptional programs through which ELA might induce brain changes. These studies have highlighted that the effects of ELA vary by brain region, cell-types and sex. Yet, while major advances were made in the past decade, the precise mechanisms through which ELA shapes the maturation and function of brain cells and their incorporation into circuits remain incompletely understood. Here, we discuss human and animal studies that focus on ELA-induced changes of the epigenome and transcriptome and explore recent technological advances that allow visualization and manipulation of neurons activated during ELA, at later stages of life. One such technology, Targeted Recombination in Active Populations (TRAP), enables precise and permanent genetic access to cells activated during specific sensitive developmental periods. Coupled with the appropriate tools, TRAP can be used to identify cellular transcriptional programs that are altered by the ELA experience in specific cell types and circuits, impacting cognitive and emotional brain functions enduringly. Understanding how ELA changes gene expression, circuit integration and function of neurons that are engaged by ELA will advance our understanding of the mechanisms employed by ELA to heighten the risk for mental illness later in life.

## 1 Introduction

Adverse experiences early in life are associated with vulnerability to cognitive and emotional dysfunction, including non-optimal cognitive performance (Kaplan et al., 2001; Fors et al., 2009; Marden et al., 2017), anxiety and augmented risk-taking (Nelson et al., 2007; Green et al., 2010; Tottenham et al., 2010; Hanson et al., 2015; Davis et al., 2017; Short and Baram, 2019). Health risk behaviors in adulthood were increased in children raised in dysfunctional households and were proportional to the degree of abuse the subjects had experienced (Felitti et al., 1998; Danese et al., 2017). Several studies also point out to alterations of the reward circuitry, emotion regulation and processing, and high risk for mood disorders such as depression, following ELA (Gaffrey et al., 2018; Kopala-Sibley et al., 2018; Novick et al., 2018; Dixon et al., 2020).

ELA is highly prevalent, posing a major social and public health challenge. A recent meta-analysis study that included almost half a million children from 18 countries, found that 6 out of 10 children experienced 1 or more adverse early life experiences (ACEs), and 15% experienced 4 or more (Madigan et al., 2025). Higher prevalence of ACEs was observed in children from low-income households, (Madigan et al., 2025; Farooq et al., 2024) or adolescents who were female, multiracial, identifying as indigenous populations or non-heterosexual (Swedo et al., 2024). Although ELA is linked to adverse physical and mental health outcomes at the population level, ACEs show weak associations with negative outcomes at the individual level (Baldwin et al., 2021) making it difficult to identify vulnerable individuals.

## 2 ELA and adverse outcomes—human studies

Whereas a large body of human work associates ELA with adverse mental health outcomes, human studies are often correlational. A unique, controlled, randomized human study investigated Romanian infants placed in institutions that then either transitioned to foster care or stayed in institutional care (Nelson et al., 2007). The cognitive outcomes of children moved to foster care were significantly higher compared to those who remained institutionalized, and this was predicated on transition to a more nurturing, enriching and stable environment by around 2 years of age. These findings support a causal role of ELA in neurodevelopmental problems and suggest the existence of a sensitive neurodevelopmental period early in postnatal life (Duncan et al., 1998; Hensch, 2004; Hackman and Farah, 2009; Gee and Cohodes, 2021; Birnie and Baram, 2025).

Thus, the literature on the link between ELA and mental and cognitive health vulnerabilities is compelling. In addition, ELA is complex and multifaceted, and different dimensions of ELA may have diverse consequences on cognitive and emotional functions later in life (Sheridan and McLaughlin, 2014). Recently, in addition to the well-studied issues of poverty, neglect, abuse and maternal depression, unpredictability of signals from the parents and the community has been found to be an understudied ELA dimension, with a major impact on memory, executive functions and depression-related symptoms such as anhedonia (Davis et al., 2017, 2019; Glynn et al., 2019, 2025; Spadoni et al., 2022).

## 3 Epigenomics and transcriptomics as a plausible mechanism—human studies

As discussed above, a large body of work links ELA with neuropsychiatric disorders later in life; however, the molecular and cellular mechanisms through which ELA leads to enduring problems remain largely unclear. One potential mechanism to explain the long-term encoding of ELA is epigenetics. Epigenetic regulation refers to the control of gene expression through potentially reversible modifications to DNA, chromatin, or associated regulatory molecules that do not alter the primary coding sequence, and serves as a key interface through which environmental factors influence genomic function (Feil and Fraga, 2012). Changes to the epigenome have thus been proposed as a potential mechanism through which ELA is persistently encoded at the molecular level, leading to lasting alterations in gene expression and increased vulnerability to adverse phenotypes (Turecki et al., 2014; Short and Baram, 2019; Ochi and Dwivedi, 2022).

DNA methylation is one of the first discovered epigenetic mechanisms and, as such, one of the most widely cited in the context of ELA. Several studies have linked ELA with altered DNA methylation of specific genes (e.g., the glucocorticoid receptor gene *NR3C1*) in both peripheral tissues and postmortem brain samples (reviewed at length in Turecki and Meaney, 2016; Cecil et al., 2020), and a few studies have investigated its impact on genome-wide DNA methylation levels (reviewed in Cecil et al., 2020; Parel and Peña, 2022). DNA methylation measured from buccal swabs or saliva has been suggested to contain signatures of ELA (Marini et al., 2019; Sumner et al., 2022; Short et al., 2024). A challenge in identifying DNA methylation signatures of ELA is due to large inter-individual variation in the human genome and hence, in DNA methylation patterns (Bock et al., 2008). This variance has hampered detection of common discernible methylation changes that associate with ELA or predict its outcomes. To minimize this challenge, recent studies have focused on assessing changes in DNA methylation over time in a single individual (a within-subject design), rather than DNA methylation at a single time point. Indeed, this approach has shown strong associations between these methylation changes and ELA (Sumner et al., 2022; Short et al., 2024). Of note, in Short et al., buccal swabs of infants were collected neonatally and at 1 year of life for each individual, and the average change in DNA methylation between these two times both reflected the degree of ELA and predicted cognitive outcome (executive control) at age 5 in a sex-dependent manner (Short et al., 2024).

Epigenetic modifications can also govern chromatin accessibility and gene transcription. Due to the technical challenges and low yield of high-quality RNA from non-invasive sampling methods such as buccal swabs, most human studies have relied on blood samples. Red blood cells have no nuclei. However, white blood cells may carry a durable signature of ELA on the transcriptome, including gene-expression profiles relating to inflammation (Dieckmann et al., 2020; Edelmann et al., 2023; Etzel et al., 2024).

Direct examinations of transcriptomic changes in postmortem brain tissue have been limited. Wang et al. found that depressed individuals had elevated expression of *CRH*, *CRHR1*, *ERa*, and *NR3C2*, as well as decreased AR in the paraventricular nucleus of the hypothalamus (Wang et al., 2008). Labonte et al. reported decreased expression of hippocampal glucocorticoid receptor variants 1B, 1C, and 1H in individuals who died by suicide with a history of abuse compared to those without abuse history (Labonte et al., 2012). Investigations into DNA methylation, histone modifications, and gene expression within the lateral amygdala of individuals who experienced ELA revealed converging evidence suggesting changes in immune related pathways (Lutz et al., 2021). Postmortem analyses of the anterior cingulate cortex by Lutz et al. revealed that suicide completers with a history of abuse exhibit altered myelin-related gene expression and methylation in oligodendrocytes (Lutz et al., 2017). Single-nuclei sequencing of the cerebral cortex of individuals with a history of childhood abuse revealed that oligodendrocyte precursor cells upregulate perineuronal net components, extracellular matrix structures that regulate neuronal plasticity, suggesting that childhood trauma may impair cortical plasticity (Tanti et al., 2021).

## 4 ELA and adverse outcomes—animal models

Human studies demonstrate associations between ELA and outcomes later in life. However, it is difficult to infer causality from such correlational studies. In addition, in human studies it is almost impossible to distinguish between genetic and environmental factors (Short and Baram, 2019; Birnie and Baram, 2025). Animal models overcome these issues: genetic variability between subjects is minimized, environmental conditions are kept stable, and, most importantly, experiments can be designed to show causality between ELA and cognitive and emotional functions later in life.

Several animal models have been developed to simulate ELA and have demonstrated cognitive deficits and altered responses to stress and to reward cues, among other outcomes. Among the most widely used are maternal separation (Biagini et al., 1998; Plotsky et al., 2005; Nishi et al., 2013), the limited bedding and nesting (LBN) model (Rice et al., 2008), and numerous related variations (Molet et al., 2014). Here we discuss LBN as a prevalent naturalistic model of ELA developed by our lab (Gilles et al., 1996; Brunson et al., 2005; Rice et al., 2008; Birnie et al., 2023) and widely adopted throughout the world (Walker et al., 2017; O’Neill et al., 2025). This simulated poverty model employs limiting the bedding and nesting materials in the rearing cages during a sensitive developmental period (Ivy et al., 2008; Molet et al., 2014; Birnie et al., 2023). Mouse or rat pups are raised in these cages from postnatal day (P) 2 to 10. During this time, maternal behavior is altered likely because of the stress induced in the dam by the limited resources (Ivy et al., 2008). Chaotic and unpredictable caring behaviors are observed, with inconsistent and fragmented bouts of care (Molet et al., 2016; Davis et al., 2017). Using this model, we showed that ELA directly causes deficits in memory (Brunson et al., 2005; Ivy et al., 2010), in a way comparable to the decreased cognitive performance observed in children raised by mothers with high unpredictability (Davis et al., 2017). Thus, the findings in experimental animals are directly relevant to humans. In addition to cognitive deficits, reward behaviors are impacted in mice and rats that are raised in this ELA model: males exhibit decreased motivation for palatable food and sex cues (Molet et al., 2016; Bolton et al., 2018b; Birnie et al., 2023; Kooiker et al., 2024). In contrast, female mice and rats exhibit increased motivation and consumption for palatable foods, sex cues, and non-natural rewards including opioids (Levis et al., 2021; Birnie et al., 2023; Kooiker et al., 2024).

## 5 Epigenomics and transcriptomics as a plausible mechanism—animal studies

A potential mechanism through which transient ELA may induce deficits later in life involves epigenetic and transcriptional reprogramming that, in turn, govern neuronal functions and responses to stimuli (Nestler, 2014; Bale, 2015; Birnie and Baram, 2025; Peña, 2025; Torres-Berrío et al., 2025). Approaches to study ELA’s epigenetic effects have included both locus-specific analyses and genome wide surveys. In targeted studies, reduced maternal care led to both DNA hypermethylation and decreased H3K9 histone acetylation at the *Nr3c1* locus in the rat hippocampus, coinciding with decreased gene expression (Weaver et al., 2004; McGowan et al., 2011).

Parallel work in the medial prefrontal cortex of rats exposed to caregiver maltreatment found sex specific methylation at the *Bdnf* locus, and female specific decrease in histone acetylation at *Bdnf* exon IV during adulthood, indicating enduring epigenetic priming established during development (Blaze et al., 2013, 2015). DNA methylation profiling of buccal-cell DNA at two timepoints from rats raised under the limited bedding and nesting paradigm revealed an epigenetic signature differentiating ELA rats from control (Jiang et al., 2019).

In addition to these persistent epigenetic alterations of single genes, ELA reconfigures large-scale transcriptional programs in key brain regions. For example, Peña et al. described, in the ventral tegmental area of mice, ELA-induced transcriptional reprogramming, and manipulation of the upstream regulator of this reprogramming modulated depression-like behaviors (Peña et al., 2017). A similar transcriptional reprogramming was found in the hippocampus of rats that experienced ELA (Bolton et al., 2020). In this study, blocking of the upstream regulator (the neuron-restrictive silencer factor or NRSF) rescued spatial memory deficits observed after ELA. Priming of sets of genes by ELA has recently been identified (Peña et al., 2019; Kos et al., 2023), which sets the stage for the differential expression of these genes only after an additional stress later in life, defining a state of ELA-induced vulnerability. While these investigations have focused on bulk changes in heterogeneous brain regions, sequencing of specific neuronal populations or single cells allowed better resolution of the epigenomic effects of ELA. For example, ELA induced gene expression changes in specific subpopulations of corticotropin-releasing hormone-expressing neurons in the hypothalamic paraventricular nucleus (Short et al., 2023), persistent epigenetic and translational changes in the stress-sensitive CA3 neuronal population (Marrocco et al., 2019) and overexpression of enzymes associated with stress-susceptibility in D2-type medium spiny neurons (Kronman et al., 2021). Even though high-throughput data studies in non-neuronal cell populations in the context of ELA are limited, there is evidence that ELA changes gene expression in microglia (Réus et al., 2019; Bolton et al., 2022), astrocytes (Abbink et al., 2020) and oligodendrocytes (Lutz et al., 2017; Treccani et al., 2021; Sharma et al., 2023).

## 6 Probing epigenomic mechanisms specifically in neurons engaged by ELA using TRAP

Studies investigating the mechanisms through which ELA might change the brain have so far focused on specific brain areas and their connections (Peña et al., 2017; Bolton et al., 2018a; Bolton et al., 2020; Birnie et al., 2023) or specific cell types (Kos et al., 2023; Short et al., 2023; Warhaftig et al., 2023). However, an additional factor that might be pertinent is the activation status of cells during the ELA period, since neuronal activity is critical to the maturation, synaptic and circuit integration, and transcriptional transitions of neurons throughout development (Reh et al., 2020; Stroud et al., 2020; Birnie and Baram, 2022). In addition, neurons that were active during specific developmental times showed hub-like connectivity and their activity led to population bursts at later developmental stages (Wang et al., 2024).

With the advent of new tools and transgenic mouse lines we can now gain permanent genetic access to neurons that were previously activated at a certain time period (Guenthner et al., 2013; DeNardo et al., 2019). This method, called Targeted Recombination in Active Populations (TRAP), relies on the rapid expression of immediate early genes (IEGs) such as *Arc* and *Fos* in activated cells (DeNardo and Luo, 2017; Franceschini et al., 2020). The timing and duration of the period is controlled through the administration of tamoxifen, which initiates a ~ 36-h time window when activated neurons undergo Cre-mediated recombination and start expressing a permanent, visible cell marker. This way TRAP allows genetic access to tagged (TRAPed) neurons that might be molecularly distinct and spatially distributed but are functionally similar or organized in ensembles (Guenthner et al., 2013; DeNardo et al., 2019; Wang et al., 2024).

In the last decade, TRAP has been improved (TRAP2 version in DeNardo et al., 2019) and used extensively for tagging and characterization of neuronal ensembles linked to specific behaviors (DeNardo et al., 2019; Herzog et al., 2020; Xing et al., 2021; Kooiker et al., 2023; Kitagawa et al., 2024), for characterizing neuronal circuits (Gongwer et al., 2023) and for circuit manipulation, by combining TRAP-mediated effector expression with opto- or chemogenetic receptors (Clawson et al., 2021; Imoto et al., 2021; Xing et al., 2021; Kitagawa et al., 2024). TRAP was recently used to permanently tag cells activated as early as embryonic day E18, and in two recent studies TRAP revealed cells that were activated during the ELA period (P2-P10 in Kooiker et al., 2023; P10-P17 in Balouek et al., 2023). Importantly, aberrant behaviors—the outcome measures of the effects of ELA on brain function—were ameliorated when the TRAPed cells were chemogenetically inhibited (Balouek et al., 2023; Kooiker et al., 2024). More specifically, in the first study, inhibition of cells activated by ELA ameliorated social avoidance following adult stress (Balouek et al., 2023), and in the second study inhibition of ELA-TRAPed cells normalized an anhedonia-like phenotype in males and a hyper-motivated phenotype in females (Kooiker et al., 2024).

**Figure 1:**
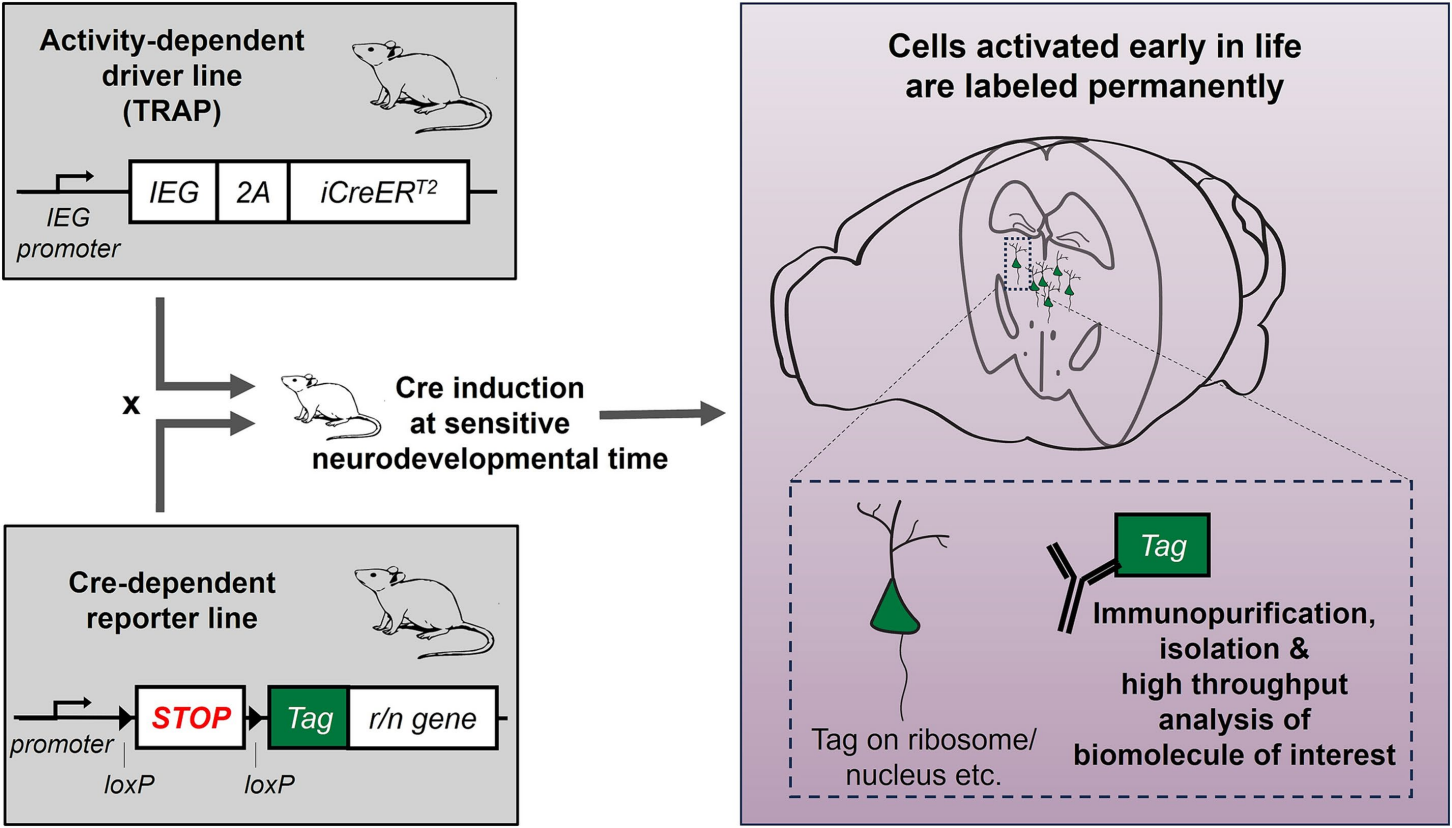
Interrogating gene expression changes selectively and specifically in neurons engaged during early-life adversity: combining activity-dependent genetic tagging with molecular tools. The activity-dependent genetic labeling approach (TRAP) allows expression of inducible CreER^T2^ recombinase under the promoter of an immediate early gene (IEG, e.g., *Fos, Arc* etc.). This mouse line is crossed with a Cre-dependent reporter line, expressing a tag (e.g., GFP, mCHERRY). In the offspring, CreER^T2^ is induced early in life, when mice are raised in typical conditions or in simulated ELA, and any cells expressing the IEG during this time will be labeled permanently with the tag. The tag can be attached to a ribosomal or nuclear protein, to allow isolation of ribosomal or nuclear RNA for high-throughput analyses.

Beyond circuit delineation and manipulation, TRAP also offers permanent access to the molecular makeup of TRAPed cells. Indeed, using immunohistochemistry, Dirven et al. recently tested for the histone modifier HDAC2 and methylation/hydroxymethylation in cells activated and TRAPed during stress, in mice that were categorized as resilient or susceptible to depression (Dirven et al., 2024). TRAPed cells have also been sorted based on the expression of their permanent tag, and their RNA was isolated and sequenced (Imoto et al., 2021). In the future, TRAP could be combined with a ribosomal or nuclear tag and translating ribosome affinity purification or nuclear affinity purification (Heiman et al., 2014; Roh et al., 2017), to provide access to the TRAPed cells’ translatome or nuclear transcriptome respectively, without the need for sorting. The expression of a tag, if strong enough, could also be used to identify TRAPed cells during the analysis of single cell or single nucleus RNA sequencing data. The idea of performing omics in populations of cells that were activated together or at the same time could help understand how stimuli or events during a specific time period (especially sensitive developmental times such as early life) affect gene expression and cell function (Figure 1). Finally, permanent genetic access can also enable targeted gene editing. One important consideration is that since the genetic labeling depends on neuronal activation and not any other specific characteristics, TRAPed cells most likely encompass subpopulations of cells with different properties. For omics studies, this heterogeneity may necessitate larger sample sizes to overcome inter-sample variability and achieve sufficient statistical power.

## 7 Conclusion

Understanding how transient ELA changes the brain enduringly requires elucidating the underlying mechanisms, most prominently, epigenetic changes in specific brain cell populations. Such studies, which are crucial for our understanding of how ELA can result in mental illness, cannot be conducted in humans, in cell lines or in organoids. Therefore, animal models are essential in the quest to identify the relevant brain circuits and molecular mechanisms bridging ELA and human disease, information that is required for designing therapies and interventions. In experimental models, we need to further embrace the complexity of the brain, and probe distinct cell populations in brain regions and circuits that execute the many types of behaviors that are impacted by ELA. The rise of new technologies is now allowing a focus on specific cell types and single cells. TRAP allows us to explore the effects of ELA taking into account another crucial factor; the cells’ activation status during a sensitive developmental time. This honed approach will eliminate dilution of engaged cells in large neuronal populations that are not influenced by ELA and hold promise to further advance our understanding of the complex, fascinating and highly important mechanisms by which ELA “gets under the skin.”

## References

[ref1] Abbink M. R. Kotah J. M. Hoeijmakers L. Mak A. Yvon-Durocher G. van der Gaag B.. (2020). Characterization of astrocytes throughout life in wildtype and APP/PS1 mice after early-life stress exposure. J. Neuroinflammation 17:91. doi: 10.1186/S12974-020-01762-Z, PMID: 32197653 PMC7083036

[ref2] Baldwin J. R. Caspi A. Meehan A. J. Ambler A. Arseneault L. Fisher H. L.. (2021). Population vs individual prediction of poor health from results of adverse childhood experiences screening. JAMA Pediatr. 175, 385–393. doi: 10.1001/JAMAPEDIATRICS.2020.5602, PMID: 33492366 PMC7835926

[ref3] Bale T. L. (2015). Epigenetic and transgenerational reprogramming of brain development. Nat. Rev. Neurosci. 16, 332–344. doi: 10.1038/nrn3818, PMID: 25921815 PMC7064155

[ref4] Balouek J. A. McLain C. A. Minerva A. R. Rashford R. L. Bennett S. N. Rogers F. D.. (2023). Reactivation of early-life stress-sensitive neuronal ensembles contributes to lifelong stress hypersensitivity. J. Neurosci. 43, 5996–6009. doi: 10.1523/JNEUROSCI.0016-23.2023, PMID: 37429717 PMC10451005

[ref5] Biagini G. Pich E. M. Carani C. Marrama P. Agnati L. F. (1998). Postnatal maternal separation during the stress hyporesponsive period enhances the adrenocortical response to novelty in adult rats by affecting feedback regulation in the CA1 hippocampal field. Int. J. Dev. Neurosci. 16, 187–197. doi: 10.1016/S0736-5748(98)00019-7, PMID: 9785115

[ref6] Birnie M. T. Baram T. Z. (2022). Principles of emotional brain circuit maturation. Science 376, 1055–1056. doi: 10.1126/science.abn4016, PMID: 35653483 PMC9840462

[ref7] Birnie M. T. Baram T. Z. (2025). The evolving neurobiology of early-life stress. Neuron 113, 1474–1490. doi: 10.1016/J.NEURON.2025.02.016, PMID: 40101719 PMC12097948

[ref8] Birnie M. T. Short A. K. de Carvalho G. B. Taniguchi L. Gunn B. G. Pham A. L.. (2023). Stress-induced plasticity of a CRH/GABA projection disrupts reward behaviors in mice. Nat. Commun. 14, 1–10. doi: 10.1038/s41467-023-36780-x, PMID: 36841826 PMC9968307

[ref9] Blaze J. Asok A. Roth T. L. (2015). Long-term effects of early-life caregiving experiences on brain-derived neurotrophic factor histone acetylation in the adult rat mPFC. *Stress* (Amsterdam, Netherlands) 18, 607–615. doi: 10.3109/10253890.2015.1071790, PMID: 26305287 PMC4879775

[ref10] Blaze J. Scheuing L. Roth T. L. (2013). Differential methylation of genes in the medial prefrontal cortex of developing and adult rats following exposure to maltreatment or nurturing care during infancy. Dev. Neurosci. 35, 306–316. doi: 10.1159/000350716, PMID: 23751776 PMC3847900

[ref11] Bock C. Walter J. Paulsen M. Lengauer T. (2008). Inter-individual variation of DNA methylation and its implications for large-scale epigenome mapping. Nucleic Acids Res. 36:e55. doi: 10.1093/NAR/GKN122, PMID: 18413340 PMC2425484

[ref12] Bolton J. L. Molet J. Regev L. Chen Y. Rismanchi N. Haddad E.. (2018a). Anhedonia following early-life adversity involves aberrant interaction of reward and anxiety circuits and is reversed by partial silencing of amygdala Corticotropin-releasing hormone gene. Biol. Psychiatry 83, 137–147. doi: 10.1016/j.biopsych.2017.08.023, PMID: 29033027 PMC5723546

[ref13] Bolton J. L. Ruiz C. M. Rismanchi N. Sanchez G. A. Castillo E. Huang J.. (2018b). Early-life adversity facilitates acquisition of cocaine self-administration and induces persistent anhedonia. Neurobiol. Stress 8, 57–67. doi: 10.1016/j.ynstr.2018.01.002 29888304 PMC5991313

[ref14] Bolton J. L. Schulmann A. Garcia-Curran M. M. Regev L. Chen Y. Kamei N.. (2020). Unexpected transcriptional programs contribute to hippocampal memory deficits and neuronal stunting after early-life adversity. Cell Rep. 33:108511. doi: 10.1016/j.celrep.2020.108511, PMID: 33326786 PMC7817243

[ref15] Bolton J. L. Short A. K. Othy S. Kooiker C. L. Shao M. Gunn B. G.. (2022). Early stress-induced impaired microglial pruning of excitatory synapses on immature CRH-expressing neurons provokes aberrant adult stress responses. Cell Rep. 38:110600. doi: 10.1016/J.CELREP.2022.110600, PMID: 35354026 PMC9014810

[ref16] Brunson K. L. Kramár E. Lin B. Chen Y. Colgin L. L. Yanagihara T. K.. (2005). Mechanisms of late-onset cognitive decline after early-life stress. J. Neurosci. 25, 9328–9338. doi: 10.1523/JNEUROSCI.2281-05.2005, PMID: 16221841 PMC3100717

[ref17] Cecil C. A. M. Zhang Y. Nolte T. (2020). Childhood maltreatment and DNA methylation: a systematic review. Neurosci. Biobehav. Rev. 112, 392–409. doi: 10.1016/J.NEUBIOREV.2020.02.019, PMID: 32081689

[ref18] Clawson B. C. Pickup E. J. Ensing A. Geneseo L. Shaver J. Gonzalez-Amoretti J.. (2021). Causal role for sleep-dependent reactivation of learning-activated sensory ensembles for fear memory consolidation. Nat. Commun. 12:1200. doi: 10.1038/S41467-021-21471-2, PMID: 33619256 PMC7900186

[ref19] Danese A. Moffitt T. E. Arseneault L. Bleiberg B. A. Dinardo P. B. Gandelman S. B.. (2017). The origins of cognitive deficits in victimized children: implications for neuroscientists and clinicians. Am. J. Psychiatry 174, 349–361. doi: 10.1176/APPI.AJP.2016.16030333, PMID: 27794691 PMC5378606

[ref20] Davis E. P. Korja R. Karlsson L. Glynn L. M. Sandman C. A. Vegetabile B.. (2019). Across continents and demographics, unpredictable maternal signals are associated with children’s cognitive function. EBioMedicine 46, 256–263. doi: 10.1016/J.EBIOM.2019.07.025, PMID: 31362905 PMC6710909

[ref21] Davis E. P. Stout S. A. Molet J. Vegetabile B. Glynn L. M. Sandman C. A.. (2017). Exposure to unpredictable maternal sensory signals influences cognitive development across species. Proc. Natl. Acad. Sci. USA 114, 10390–10395. doi: 10.1073/pnas.1703444114, PMID: 28893979 PMC5625898

[ref22] DeNardo L. A. Liu C. D. Allen W. E. Adams E. L. Friedmann D. Fu L.. (2019). Temporal evolution of cortical ensembles promoting remote memory retrieval. Nat. Neurosci. 22, 460–469. doi: 10.1038/s41593-018-0318-7, PMID: 30692687 PMC6387639

[ref23] DeNardo L. A. Luo L. (2017). Genetic strategies to access activated neurons. Curr. Opin. Neurobiol. 45, 121–129. doi: 10.1016/J.CONB.2017.05.014, PMID: 28577429 PMC5810937

[ref24] Dieckmann L. Cole S. Kumsta R. (2020). Stress genomics revisited: gene co-expression analysis identifies molecular signatures associated with childhood adversity. Transl. Psychiatry 10, 1–11. doi: 10.1038/S41398-020-0730-0 32066736 PMC7026041

[ref25] Dirven B. C. J. van Melis L. Daneva T. Dillen L. Homberg J. R. Kozicz T.. (2024). Hippocampal trauma memory processing conveying susceptibility to traumatic stress. Neuroscience 540, 87–102. doi: 10.1016/j.neuroscience.2024.01.007, PMID: 38220126

[ref26] Dixon R. Wu S. Emile A. Shores R. Lofaro O. Anacker C. (2020). Early life stress causes neurobiological and physiological impairments that precede behavioral despair in adulthood. Biol. Psychiatry 87, S386–S387. doi: 10.1016/J.BIOPSYCH.2020.02.989

[ref27] Duncan G. J. Brooks-Gunn J. Yeung W. Smith J. R. (1998). How much does childhood poverty affect the life chances of children? Am. Sociol. Rev. 63, 406–423. doi: 10.2307/2657556

[ref28] Edelmann S. Wiegand A. Hentrich T. Pasche S. Schulze-Hentrich J. M. Munk M. H. J.. (2023). Blood transcriptome analysis suggests an indirect molecular association of early life adversities and adult social anxiety disorder by immune-related signal transduction. Front. Psych. 14:1125553. doi: 10.3389/fpsyt.2023.1125553, PMID: 37181876 PMC10168183

[ref29] Etzel L. Apsley A. T. Hastings W. J. Ye Q. Shalev I. (2024). Early life adversity is associated with differential gene expression in immune cells: a cluster-based analysis across an acute psychosocial stressor. Brain Behav. Immun. 119, 724–733. doi: 10.1016/J.BBI.2024.04.035, PMID: 38663776 PMC11190835

[ref30] Farooq B. Allen K. Russell A. E. Howe L. D. Mars B. (2024). The association between poverty and longitudinal patterns of adverse childhood experiences across childhood and adolescence: findings from a prospective population-based cohort study in the UK. Child Abuse Negl. 156:107014. doi: 10.1016/J.CHIABU.2024.107014, PMID: 39232377

[ref31] Feil R. Fraga M. F. (2012). Epigenetics and the environment: emerging patterns and implications. Nat. Rev. Genet. 13, 97–109. doi: 10.1038/NRG3142, PMID: 22215131

[ref32] Felitti V. J. Anda R. F. Nordenberg D. Williamson D. F. Spitz A. M. Edwards V.. (1998). Relationship of childhood abuse and household dysfunction to many of the leading causes of death in adults: the adverse childhood experiences (ACE) study. Am. J. Prev. Med. 14, 245–258. doi: 10.1016/S0749-3797(98)00017-8, PMID: 9635069

[ref33] Fors S. Lennartsson C. Lundberg O. (2009). Childhood living conditions, socioeconomic position in adulthood, and cognition in later life: exploring the associations. J. Gerontol. B Psychol. Sci. Soc. Sci. 64, 750–757. doi: 10.1093/GERONB/GBP029, PMID: 19420323

[ref34] Franceschini A. Costantini I. Pavone F. S. Silvestri L. (2020). Dissecting neuronal activation on a brain-wide scale with immediate early genes. Front. Neurosci. 14:569517. doi: 10.3389/fnins.2020.569517, PMID: 33192255 PMC7645181

[ref35] Gaffrey M. S. Barch D. M. Bogdan R. Farris K. Petersen S. E. Luby J. L. (2018). Amygdala reward reactivity mediates the association between preschool stress response and depression severity. Biol. Psychiatry 83, 128–136. doi: 10.1016/J.BIOPSYCH.2017.08.020, PMID: 29102026 PMC5723551

[ref36] Gee D. G. Cohodes E. M. (2021). Influences of caregiving on development: a sensitive period for biological embedding of predictability and safety cues. Curr. Dir. Psychol. Sci. 30, 376–383. doi: 10.1177/09637214211015673, PMID: 34675455 PMC8528226

[ref37] Gilles E. E. Schultz L. Baram T. Z. (1996). Abnormal corticosterone regulation in an immature rat model of continuous chronic stress. Pediatr. Neurol. 15, 114–119. doi: 10.1016/0887-8994(96)00153-1, PMID: 8888044 PMC3415889

[ref38] Glynn L. M. Liu S. R. Golden C. Weiss M. Lucas C. T. Cooper D. M.. (2025). Contribution of an under-recognized adversity to child health risk: large-scale, population-based ACEs screening. MedRxiv:2025.02.04.25321682. doi: 10.1101/2025.02.04.25321682, PMID: 39974059 PMC11838625

[ref39] Glynn L. M. Stern H. S. Howland M. A. Risbrough V. B. Baker D. G. Nievergelt C. M.. (2019). Measuring novel antecedents of mental illness: the questionnaire of unpredictability in childhood. Neuropsychopharmacology 44, 876–882. doi: 10.1038/S41386-018-0280-9, PMID: 30470840 PMC6461958

[ref40] Gongwer M. W. Klune C. B. Couto J. Jin B. Enos A. S. Chen R.. (2023). Brain-wide projections and differential encoding of prefrontal neuronal classes underlying learned and innate threat avoidance. J. Neurosci. 43, 5810–5830. doi: 10.1523/JNEUROSCI.0697-23.2023, PMID: 37491314 PMC10423051

[ref41] Green J. G. McLaughlin K. A. Berglund P. A. Gruber M. J. Sampson N. A. Zaslavsky A. M.. (2010). Childhood adversities and adult psychiatric disorders in the National Comorbidity Survey Replication I: associations with first onset of DSM-IV disorders. Arch. Gen. Psychiatry 67, 113–123. doi: 10.1001/ARCHGENPSYCHIATRY.2009.186, PMID: 20124111 PMC2822662

[ref42] Guenthner C. J. Miyamichi K. Yang H. H. Heller H. C. Luo L. (2013). Permanent genetic access to transiently active neurons via TRAP: targeted recombination in active populations. Neuron 78, 773–784. doi: 10.1016/j.neuron.2013.03.025, PMID: 23764283 PMC3782391

[ref43] Hackman D. A. Farah M. J. (2009). Socioeconomic status and the developing brain. Trends Cogn. Sci. 13, 65–73. doi: 10.1016/J.TICS.2008.11.003, PMID: 19135405 PMC3575682

[ref44] Hanson J. L. Hariri A. R. Williamson D. E. (2015). Blunted ventral striatum development in adolescence reflects emotional neglect and predicts depressive symptoms. Biol. Psychiatry 78, 598–605. doi: 10.1016/j.biopsych.2015.05.010, PMID: 26092778 PMC4593720

[ref45] Heiman M. Kulicke R. Fenster R. J. Greengard P. Heintz N. (2014). Cell type-specific mRNA purification by translating ribosome affinity purification (TRAP). Nat. Protoc. 9, 1282–1291. doi: 10.1038/nprot.2014.085, PMID: 24810037 PMC4102313

[ref46] Hensch T. K. (2004). Critical period regulation. Annu. Rev. Neurosci. 27, 549–579. doi: 10.1146/ANNUREV.NEURO.27.070203.144327 15217343

[ref47] Herzog D. P. Mellema R. M. Remmers F. Lutz B. Müller M. B. Treccani G. (2020). Sexually dimorphic behavioral profile in a transgenic model enabling targeted recombination in active neurons in response to ketamine and (2R,6R)-hydroxynorketamine administration. Int. J. Mol. Sci. 21:2142. doi: 10.3390/IJMS21062142, PMID: 32244978 PMC7139539

[ref48] Imoto D. Yamamoto I. Matsunaga H. Yonekura T. Lee M. L. Kato K. X.. (2021). Refeeding activates neurons in the dorsomedial hypothalamus to inhibit food intake and promote positive valence. Mol. Metab. 54:101366. doi: 10.1016/j.molmet.2021.101366, PMID: 34728342 PMC8609163

[ref49] Ivy A. S. Brunson K. L. Sandman C. Baram T. Z. (2008). Dysfunctional nurturing behavior in rat dams with limited access to nesting material: a clinically relevant model for early-life stress. Neuroscience 154, 1132–1142. doi: 10.1016/J.NEUROSCIENCE.2008.04.019, PMID: 18501521 PMC2517119

[ref50] Ivy A. S. Rex C. S. Chen Y. Dubé C. Maras P. M. Grigoriadis D. E.. (2010). Hippocampal dysfunction and cognitive impairments provoked by chronic early-life stress involve excessive activation of CRH receptors. J. Neurosci. 30, 13005–13015. doi: 10.1523/JNEUROSCI.1784-10.2010, PMID: 20881118 PMC2991143

[ref51] Jiang S. Kamei N. Bolton J. L. Ma X. Stern H. S. Baram T. Z.. (2019). Intra-individual methylomics detects the impact of early-life adversity. Life Sci. Alliance 2:e201800204. doi: 10.26508/LSA.201800204, PMID: 30936186 PMC6445397

[ref52] Kaplan G. A. Turrell G. Lynch J. W. Everson S. A. Helkala E. L. Salonen J. T. (2001). Childhood socioeconomic position and cognitive function in adulthood. Int. J. Epidemiol. 30, 256–263. doi: 10.1093/IJE/30.2.256, PMID: 11369724

[ref53] Kitagawa K. Takemoto T. Seiriki K. Kasai A. Hashimoto H. Nakazawa T. (2024). Socially activated neurons in the anterior cingulate cortex are essential for social behavior in mice. Biochem. Biophys. Res. Commun. 726:150251. doi: 10.1016/j.bbrc.2024.150251, PMID: 38936249

[ref54] Kooiker C. L. Birnie M. T. Floriou-Servou A. Ding Q. Thiagarajan N. Hardy M.. (2024). Paraventricular thalamus neuronal ensembles encode early-life adversity and mediate the consequent sex-dependent disruptions of adult reward behaviors. BioRxiv. doi: 10.1101/2024.04.28.591547

[ref55] Kooiker C. L. Chen Y. Birnie M. T. Baram T. Z. (2023). Genetic tagging uncovers a robust, selective activation of the thalamic paraventricular nucleus by adverse experiences early in life. Biol. Psychiatry Glob. Open Sci. 3, 746–755. doi: 10.1016/j.bpsgos.2023.01.002, PMID: 37881549 PMC10593902

[ref56] Kopala-Sibley D. C. Cyr M. Finsaas M. C. Orawe J. Huang A. Tottenham N.. (2018). Early childhood parenting predicts late childhood brain functional connectivity during emotion perception and reward processing. Child Dev. 91, 110–128. doi: 10.1111/cdev.13126, PMID: 30102429 PMC6374219

[ref57] Kos A. Lopez J. P. Bordes J. de Donno C. Dine J. Brivio E.. (2023). Early life adversity shapes social subordination and cell type-specific transcriptomic patterning in the ventral hippocampus. Sci. Adv. 9:eadj3793. doi: 10.1126/SCIADV.ADJ3793/SUPPL_FILE/SCIADV.ADJ3793_SM.PDF, PMID: 38039370 PMC10691768

[ref58] Kronman H. Torres-Berrío A. Sidoli S. Issler O. Godino A. Ramakrishnan A.. (2021). Long-term behavioral and cell-type-specific molecular effects of early life stress are mediated by H3K79me2 dynamics in medium spiny neurons. Nat. Neurosci. 24, 667–676. doi: 10.1038/s41593-021-00814-8, PMID: 33723435 PMC8216773

[ref59] Labonte B. Yerko V. Gross J. Mechawar N. Meaney M. J. Szyf M.. (2012). Differential glucocorticoid receptor exon 1B, 1C, and 1H expression and methylation in suicide completers with a history of childhood abuse. Biol. Psychiatry 72, 41–48. doi: 10.1016/J.BIOPSYCH.2012.01.034, PMID: 22444201

[ref60] Levis S. C. Bentzley B. S. Molet J. Bolton J. L. Perrone C. R. Baram T. Z.. (2021). On the early life origins of vulnerability to opioid addiction. Mol. Psychiatry 26, 4409–4416. doi: 10.1038/s41380-019-0628-5, PMID: 31822817 PMC7282971

[ref61] Lutz P. E. Chay M. A. Pacis A. Chen G. G. Aouabed Z. Maffioletti E.. (2021). Non-CG methylation and multiple histone profiles associate child abuse with immune and small GTPase dysregulation. Nat. Commun. 12, 1132–1116. doi: 10.1038/s41467-021-21365-3, PMID: 33602921 PMC7892573

[ref62] Lutz P.-E. Tanti A. Gasecka A. Barnett-Burns S. Kim J. J. Zhou Y.. (2017). Association of a history of child abuse with impaired myelination in the anterior cingulate cortex: convergent epigenetic, transcriptional, and morphological evidence. Am. J. Psychiatry 174, 1185–1194. doi: 10.1176/appi.ajp.2017.16111286 28750583

[ref63] Madigan S. Thiemann R. Deneault A. A. Fearon R. M. P. Racine N. Park J.. (2025). Prevalence of adverse childhood experiences in child population samples: a systematic review and meta-analysis. JAMA Pediatr. 179, 19–33. doi: 10.1001/JAMAPEDIATRICS.2024.4385, PMID: 39527072 PMC11555579

[ref64] Marden J. R. Tchetgen Tchetgen E. J. Kawachi I. Glymour M. M. (2017). Contribution of socioeconomic status at 3 life-course periods to late-life memory function and decline: early and late predictors of dementia risk. Am. J. Epidemiol. 186, 805–814. doi: 10.1093/aje/kwx155, PMID: 28541410 PMC5859987

[ref65] Marini S. Davis K. A. Soare T. W. Zhu Y. Suderman M. J. Simpkin A. J.. (2019). Adversity exposure during sensitive periods predicts accelerated epigenetic aging in children. Psychoneuroendocrinology 113:104484. doi: 10.1016/J.PSYNEUEN.2019.104484 31918390 PMC7832214

[ref66] Marrocco J. Gray J. D. Kogan J. F. Einhorn N. R. O’Cinneide E. M. Rubin T. G.. (2019). Early life stress restricts translational reactivity in CA3 neurons associated with altered stress responses in adulthood. Front. Behav. Neurosci. 13:157. doi: 10.3389/fnbeh.2019.00157, PMID: 31354448 PMC6637287

[ref67] McGowan P. O. Suderman M. Sasaki A. Huang T. C. T. Hallett M. Meaney M. J.. (2011). Broad epigenetic signature of maternal Care in the Brain of adult rats. PLoS One 6:e14739. doi: 10.1371/JOURNAL.PONE.0014739, PMID: 21386994 PMC3046141

[ref68] Molet J. Heins K. Zhuo X. Mei Y. T. Regev L. Baram T. Z.. (2016). Fragmentation and high entropy of neonatal experience predict adolescent emotional outcome. Transl. Psychiatry 6:e702. doi: 10.1038/tp.2015.200, PMID: 26731439 PMC5068874

[ref69] Molet J. Maras P. M. Avishai-Eliner S. Baram T. Z. (2014). Naturalistic rodent models of chronic early-life stress. Dev. Psychobiol. 56, 1675–1688. doi: 10.1002/DEV.21230, PMID: 24910169 PMC4777289

[ref70] Nelson C. A. Zeanah C. H. Fox N. A. Marshall P. J. Smyke A. T. Guthrie D. (2007). Cognitive recovery in socially deprived young children: the Bucharest early intervention project. Science 318, 1937–1940. doi: 10.1126/science.1143921 18096809

[ref71] Nestler E. J. (2014). Epigenetic mechanisms of depression. JAMA Psychiatr. 71, 454–456. doi: 10.1001/JAMAPSYCHIATRY.2013.4291, PMID: 24499927 PMC4057796

[ref72] Nishi M. Horii-Hayashi N. Sasagawa T. Matsunaga W. (2013). Effects of early life stress on brain activity: implications from maternal separation model in rodents. Gen. Comp. Endocrinol. 181, 306–309. doi: 10.1016/J.YGCEN.2012.09.024, PMID: 23032077

[ref73] Novick A. M. Levandowski M. L. Laumann L. E. Philip N. S. Price L. H. Tyrka A. R. (2018). The effects of early life stress on reward processing. J. Psychiatr. Res. 101, 80–103. doi: 10.1016/J.JPSYCHIRES.2018.02.002, PMID: 29567510 PMC5889741

[ref74] O’Neill O. S. Terstege D. J. Gill A. K. Edge-Partington M. Ramkumar R. Epp J. R.. (2025). An open-source and highly adaptable rodent limited bedding and nesting apparatus for chronic early life stress. Eneuro 12:ENEURO.0081-25.2025. doi: 10.1523/ENEURO.0081-25.2025, PMID: 40555522 PMC12203765

[ref75] Ochi S. Dwivedi Y. (2022). Dissecting early life stress-induced adolescent depression through epigenomic approach. Mol. Psychiatry 28, 141–153. doi: 10.1038/S41380-022-01907-X 36517640 PMC9812796

[ref76] Parel S. T. Peña C. J. (2022). Genome-wide signatures of early-life stress: influence of sex. Biol. Psychiatry 91, 36–42. doi: 10.1016/j.biopsych.2020.12.010, PMID: 33602500 PMC8791071

[ref77] Peña C. J. (2025). Early-life stress sensitizes response to future stress: evidence and mechanisms. Neurobiol. Stress 35:100716. doi: 10.1016/J.YNSTR.2025.100716, PMID: 40134543 PMC11932861

[ref78] Peña C. J. Kronman H. G. Walker D. M. Cates H. M. Bagot R. C. Purushothaman I.. (2017). Early life stress confers lifelong stress susceptibility in mice via ventral tegmental area OTX2. Science 356, 1185–1188. doi: 10.1126/science.aan4491, PMID: 28619944 PMC5539403

[ref79] Peña C. J. Smith M. Ramakrishnan A. Cates H. M. Bagot R. C. Kronman H. G.. (2019). Early life stress alters transcriptomic patterning across reward circuitry in male and female mice. Nat. Commun. 10:5098. doi: 10.1038/S41467-019-13085-6, PMID: 31704941 PMC6841985

[ref80] Plotsky P. M. Thrivikraman K. v. Nemeroff C. B. Caldji C. Sharma S. Meaney M. J. (2005). Long-term consequences of neonatal rearing on central corticotropin- releasing factor systems in adult male rat offspring. Neuropsychopharmacology 30, 2192–2204. doi: 10.1038/sj.npp.1300769, PMID: 15920504

[ref81] Reh R. K. Dias B. G. Nelson C. A. Kaufer D. Werker J. F. Kolbh B.. (2020). Critical period regulation across multiple timescales. Proc. Natl. Acad. Sci. USA 117:23242. doi: 10.1073/PNAS.1820836117 32503914 PMC7519216

[ref82] Réus G. Z. Silva R. H. de Moura A. B. Presa J. F. Abelaira H. M. Abatti M.. (2019). Early maternal deprivation induces microglial activation, alters glial fibrillary acidic protein immunoreactivity and indoleamine 2,3-dioxygenase during the development of offspring rats. Mol. Neurobiol. 56, 1096–1108. doi: 10.1007/S12035-018-1161-2, PMID: 29873040

[ref83] Rice C. J. Sandman C. A. Lenjavi M. R. Baram T. Z. (2008). A novel mouse model for acute and long-lasting consequences of early life stress. Endocrinology 149, 4892–4900. doi: 10.1210/EN.2008-0633, PMID: 18566122 PMC2582918

[ref84] Roh H. C. Tsai L. T. Y. Lyubetskaya A. Tenen D. Kumari M. Rosen E. D. (2017). Simultaneous transcriptional and Epigenomic profiling from specific cell types within heterogeneous tissues *in vivo*. Cell Rep. 18, 1048–1061. doi: 10.1016/j.celrep.2016.12.087, PMID: 28122230 PMC5291126

[ref85] Sharma S. Ma W. Ressler K. J. Anderson T. Li D. C. Jin P.. (2023). Dysregulation of prefrontal oligodendrocyte lineage cells across mouse models of adversity and human major depressive disorder. BioRxiv. doi: 10.1101/2023.03.09.531989

[ref86] Sheridan M. A. McLaughlin K. A. (2014). Dimensions of early experience and neural development: deprivation and threat. Trends Cogn. Sci. 18, 580–585. doi: 10.1016/J.TICS.2014.09.001, PMID: 25305194 PMC4252647

[ref87] Short A. K. Baram T. Z. (2019). Early-life adversity and neurological disease: age-old questions and novel answers. Nat. Rev. Neurol. 15, 657–669. doi: 10.1038/s41582-019-0246-5, PMID: 31530940 PMC7261498

[ref88] Short A. K. Thai C. W. Chen Y. Kamei N. Pham A. L. Birnie M. T.. (2023). Single-cell transcriptional changes in hypothalamic corticotropin-releasing factor–expressing neurons after early-life adversity inform enduring alterations in vulnerabilities to stress. Biol. Psychiatry Glob. Open Sci. 3, 99–109. doi: 10.1016/j.bpsgos.2021.12.006, PMID: 36712559 PMC9874075

[ref89] Short A. K. Weber R. Kamei N. Wilcox Thai C. Arora H. Mortazavi A.. (2024). Individual longitudinal changes in DNA-methylome identify signatures of early-life adversity and correlate with later outcome. Neurobiol. Stress 31:100652. doi: 10.1016/J.YNSTR.2024.100652, PMID: 38962694 PMC11219970

[ref90] Spadoni A. D. Vinograd M. Cuccurazzu B. Torres K. Glynn L. M. Davis E. P.. (2022). Contribution of early-life unpredictability to neuropsychiatric symptom patterns in adulthood. Depress. Anxiety 39, 706–717. doi: 10.1002/DA.23277, PMID: 35833573 PMC9881456

[ref91] Stroud H. Yang M. G. Tsitohay Y. N. Davis C. P. Sherman M. A. Hrvatin S.. (2020). An activity-mediated transition in transcription in early postnatal neurons. Neuron 107, 874–890.e8. doi: 10.1016/j.neuron.2020.06.008, PMID: 32589877 PMC7486250

[ref92] Sumner J. A. Gambazza S. Gao X. Baccarelli A. A. Uddin M. McLaughlin K. A. (2022). Epigenetics of early-life adversity in youth: cross-sectional and longitudinal associations. Clin. Epigenetics 14:48. doi: 10.1186/S13148-022-01269-9, PMID: 35395780 PMC8994405

[ref93] Swedo E. A. Niolon P. H. Anderson K. N. Li J. Brener N. Mpofu J.. (2024). Prevalence of adverse childhood experiences among adolescents. Pediatrics 154:e2024066633. doi: 10.1542/PEDS.2024-066633, PMID: 39463258 PMC11756604

[ref94] Tanti A. Belliveau C. Nagy C. Maitra M. Denux F. Perlman K.. (2021). Child abuse associates with increased recruitment of perineuronal nets in the ventromedial prefrontal cortex: a possible implication of oligodendrocyte progenitor cells. Mol. Psychiatry 27, 1552–1561. doi: 10.1038/s41380-021-01372-y, PMID: 34799691 PMC9095471

[ref95] Torres-Berrío A. Bortolami A. Peña C. J. Nestler E. J. (2025). Neurobiology of resilience to early life stress. Neuropsychopharmacology. doi: 10.1038/S41386-025-02158-4, PMID: 40562842 PMC12618491

[ref96] Tottenham N. Hare T. A. Quinn B. T. McCarry T. W. Nurse M. Gilhooly T.. (2010). Prolonged institutional rearing is associated with atypically large amygdala volume and difficulties in emotion regulation. Dev. Sci. 13, 46–61. doi: 10.1111/J.1467-7687.2009.00852.X, PMID: 20121862 PMC2817950

[ref97] Treccani G. Yigit H. Lingner T. Schleuβner V. Mey F. van der Kooij M. A.. (2021). Early life adversity targets the transcriptional signature of hippocampal NG2+ glia and affects voltage gated sodium (Nav) channels properties. Neurobiol. Stress 15:100338. doi: 10.1016/J.YNSTR.2021.100338, PMID: 34095364 PMC8164094

[ref98] Turecki G. Meaney M. J. (2016). Effects of the social environment and stress on glucocorticoid receptor gene methylation: a systematic review. Biol. Psychiatry 79, 87–96. doi: 10.1016/J.BIOPSYCH.2014.11.022, PMID: 25687413 PMC4466091

[ref99] Turecki G. Ota V. K. Belangero S. I. Jackowski A. Kaufman J. (2014). Early life adversity, genomic plasticity, and psychopathology. Lancet Psychiatry 1, 461–466. doi: 10.1016/S2215-0366(14)00022-4, PMID: 26361201 PMC5293546

[ref100] Walker C. D. Bath K. G. Joels M. Korosi A. Larauche M. Lucassen P. J.. (2017). Chronic early life stress induced by limited bedding and nesting (LBN) material in rodents: critical considerations of methodology, outcomes and translational potential. Stress 20, 421–448. doi: 10.1080/10253890.2017.1343296, PMID: 28617197 PMC5705407

[ref101] Wang S. S. Kamphuis W. Huitinga I. Zhou J. N. Swaab D. F. (2008). Gene expression analysis in the human hypothalamus in depression by laser microdissection and real-time PCR: the presence of multiple receptor imbalances. Mol. Psychiatry 13, 786–799. doi: 10.1038/mp.2008.38 18427561

[ref102] Wang D. C. Santos-Valencia F. Song J. H. Franks K. M. Luo L. (2024). Embryonically active piriform cortex neurons promote intracortical recurrent connectivity during development. Neuron 112, 2938–2954.e6. doi: 10.1016/j.neuron.2024.06.007, PMID: 38964330 PMC11377168

[ref103] Warhaftig G. Almeida D. Turecki G. (2023). Early life adversity across different cell- types in the brain. Neurosci. Biobehav. Rev. 148:105113. doi: 10.1016/J.NEUBIOREV.2023.105113, PMID: 36863603

[ref104] Weaver I. C. G. Cervoni N. Champagne F. A. D’Alessio A. C. Sharma S. Seckl J. R.. (2004). Epigenetic programming by maternal behavior. Nat. Neurosci. 7, 847–854. doi: 10.1038/NN1276 15220929

[ref105] Xing B. Mack N. R. Guo K. M. Zhang Y. X. Ramirez B. Yang S. S.. (2021). A subpopulation of prefrontal cortical neurons is required for social memory. Biol. Psychiatry 89, 521–531. doi: 10.1016/J.BIOPSYCH.2020.08.023, PMID: 33190846 PMC7867585

